# Inattentive and hyperactive traits differentially associate with interindividual functional synchrony during video viewing in young children without ADHD

**DOI:** 10.1093/texcom/tgac011

**Published:** 2022-02-28

**Authors:** Ryann Tansey, Kirk Graff, Christiane S Rohr, Dennis Dimond, Amanda Ip, Deborah Dewey, Signe Bray

**Affiliations:** 1 Child and Adolescent Imaging Research Program, University of Calgary, 28 Oki Drive NW, Calgary, AB, T3B 6A8, Canada; 2 Alberta Children’s Hospital Research Institute, University of Calgary, 28 Oki Drive NW, Calgary, AB, T3B 6A8, Canada; 3 Hotchkiss Brain Institute, University of Calgary, 3330 Hospital Drive NW, Calgary, AB, T2N 4N1, Canada; 4 Department of Pediatrics, Cumming School of Medicine, University of Calgary, 28 Oki Drive NW, Calgary, AB, T3B 6A8, Canada; 5 Department of Community Health Science, Cumming School of Medicine, University of Calgary, 3280 Hospital Drive NW, Calgary, AB, T2N 4Z6, Canada; 6 Department of Radiology, Cumming School of Medicine, University of Calgary, 3330 Hospital Drive NW, Calgary, AB, T2N 4N1, Canada

**Keywords:** developmental neuroimaging, fMRI, hyperactivity, inattention, naturalistic paradigm

## Abstract

Inattention and hyperactivity present on a spectrum and may influence the way children perceive and interact with the world. We investigated whether normative variation in inattentive and hyperactive traits was associated with differences in brain function, while children watched clips from an age-appropriate television program. Functional magnetic resonance imaging (fMRI) data and parent reports of inattention and hyperactivity traits were collected from 81 children 4–7 years of age with no parent-reported diagnoses. Data were analyzed using intersubject correlations (ISCs) in mixed effects models to determine if inattentive and hyperactive traits were associated with idiosyncrasy of fMRI response to the video. We hypothesized that pairs of children with higher average inattention and hyperactivity scores would show less interindividual brain synchrony to one another than pairs with lower average scores on these traits. Video watching engaged widespread visual, auditory, default mode and dorsal prefrontal regions. Inattention and hyperactivity were separably associated with ISC in many of these regions. Our findings suggest that the spectrum of inattention and hyperactivity traits in children without ADHD are differentially associated with neural processing of naturalistic video stimuli, which may have implications for understanding how children with different levels of these traits process audiovisual information in unconstrained conditions.

## Introduction

Inattention and hyperactivity are among the most common neurodevelopmental challenges affecting children. In children, nonclinical levels of inattention and hyperactivity have been associated with poorer outcomes such as higher rates of grade retention and graduation failure in adolescence (Bussing et al. 2010), less positive relationships with friends and parents (Rielly et al. 2006), and worse executive functioning (Brown and Casey 2016). Population-based studies have further shown that increased inattention symptoms in childhood are associated with lower academic performance in adolescence (Sayal et al. 2015; Salla et al. 2016) and reduced financial earnings in adulthood (Vergunst et al. 2019).

In adults without attention deficit/hyperactivity disorder (ADHD), inattentive and hyperactive/impulsive traits have been associated with interindividual synchrony of blood oxygen level dependent (BOLD) signal during viewing of a naturalistic movie stimuli (Salmi et al. 2020). This suggests that inattention and hyperactivity may impact the way an individual perceives and interacts with their environment. However, the relationships between inattentive and hyperactive traits and brain function in typically developing children remain understudied. Here, we investigate whether inattentive and hyperactive traits in young children without ADHD are associated with neural processing of a complex audiovisual stimulus: clips from an age-appropriate television program.

To capture individual differences in the way the brain processes audiovisual (AV) media, we used intersubject correlation (ISC; Hasson et al. 2004). Functional magnetic resonance imaging (fMRI) studies show that naturalistic AV stimuli evoke synchronized brain activity across individuals (Hasson et al. 2004, 2008; Nastase et al. 2019) in visual, auditory, emotional, navigation, and language processing regions, as well as areas related to attentional control (Bottenhorn et al. 2018). By calculating the ISC (also referred to as “interindividual synchrony”; measured as the Pearson correlation between the BOLD time courses from corresponding voxels of individuals watching the same movie), we can quantify whether individuals with similar behavioral traits show synchronized processing of an AV stimulus. Using ISC, researchers have found that individuals with autism and elevated depressive symptoms can show idiosyncratic neural responses to movies (Byrge et al. 2015; Gruskin et al. 2020). Pairwise ISC measures allow researchers to investigate idiosyncrasy and granular individual differences of both brain function and behavior, as they can capture the ways in which 2 individuals may differ in their processing of a stimulus based on their specific continuous traits and phenotypes (Finn et al. 2020).

Previous work has examined the association between a clinical diagnosis of ADHD and ISC during video viewing. In 1 adult study (Salmi et al. 2020), controls displayed more synchronized brain activity than ADHD individuals in the lateral and medial occipital cortex, precuneus, temporoparietal junction, superior temporal cortex, and—when speech or music distractors were added to the movie—the posterior parietal cortex. However, they also found that in the control group, similarity of impulsivity scores was associated with greater ISC in the cuneus, dorsomedial prefrontal cortex, and temporoparietal junction, while similarity of inattention was associated with ISC in a small bilateral region of the precuneus. Contrary to these findings, in a case–control study that compared children with ADHD to non-ADHD controls (Tang et al. 2019), the brain activity of the ADHD group was more synchronized than the control group in widespread areas of the occipital and temporal lobes. To our knowledge, the specific dimensional relationships between inattention and hyperactivity in young children without ADHD and ISCs have not yet been investigated.

Video-watching offers attractive benefits in developmental neuroimaging research, as it probes brain function in a dynamic, multimodal, and arguably more “ecologically valid” context than traditional task-based or resting-state paradigms (Sonkusare et al. 2019), opening up an entirely new realm of unique questions and techniques. It also has the added benefit of reducing head motion of young children during MRI scans (Vanderwal et al. 2015; Greene et al. 2018). Furthermore, the centrality of screen media in many children’s lives warrants investigation into it the ways it is processed by the brain and could offer important nuance regarding the potential benefits and disadvantages of this activity.

In this study, we investigate whether inattentive and hyperactive traits are associated with interindividual synchrony in young children without ADHD during the presentation of video clips from an educational television show. We hypothesized that greater inattention and hyperactivity scores would be associated with lower pairwise synchrony. Both inattention (Jonkman et al. 2017; Arabaci and Parris 2018) and hyperactivity traits (Arabaci and Parris 2018) have been positively linked to mind-wandering in children and adults without ADHD (Frick et al. 2020), which could potentially direct attention away from the shared video stimulus, resulting in decreased synchrony (Nastase et al. 2019). Specifically, we test the hypothesis that brain function is more idiosyncratic in children with higher inattention and hyperactivity trait levels.

## Materials and methods

### Participants, study procedure, and stimuli

Participants were recruited from Calgary and the surrounding area in Southern Alberta as part of a longitudinal neuroimaging study in early childhood. Study procedures were approved by the University of Calgary Conjoint Health Research Ethics Board. Parents provided informed consent for their child’s participation and children provided assent. All data collection occurred at the Alberta Children’s Hospital. Children with a history of psychiatric or neurodevelopmental disorders were excluded, as were any children with a medical condition or other contraindications that prevented participation in an MRI scan. Prior to the scan, all participants underwent a mock scanner training session to prepare them for data collection. A total of 135 children (79 females, 56 males) aged 4–7 years participated. Structural MRI, 18-min of video-watching fMRI, and parent-report behavioral data were collected. Following data collection, children were excluded from analysis if they had more than 2 min of motion corrupted volumes (motion corruption defined as >0.2-mm framewise displacement, Jenkinson criteria; Jenkinson et al. 2002). This exclusion criterion was used to minimize differences in head motion among the participants, as head motion is very high in children in the scanner (Dosenbach et al. 2017). Furthermore, in ISC analysis, which considers participants as pairs, motion is additive within each pair. In total, 54 of the original sample of 135 children were excluded (F = 28, M = 26), leading to a final sample size of *n* = 81 (F = 51, M = 30). The demographic characteristics of this final sample are presented in Table 1. The demographics of included vs. excluded children can be found in Supplementary Table S1.

**Table 1:** Demographics.

	Age (years)	Censored volumes	Average relative FD (mm)	FSIQ	SNAP-I	SNAP-H	Sex
Range	4.14–7.89	1–57	0.035–0.17	80–139	0–2.89	0–2.33	F = 51 M = 30
Mean	5.88	21.32	0.076	111.1	0.73	0.76	
SD	0.94	16.69	0.030	12.77	0.49	0.59	
Median	5.88	16.00	0.068	112	0.67	0.67	

Demographic summary for the entire sample (*n* = 81).

During video-watching fMRI, participants passively viewed a selection of clips from the children’s television show “Elmo’s World.” This stimulus was selected as it contains content that is educational and gender neutral, human faces, depicts social interaction, and is similar to the naturalistic stimuli used in other developmental neuroimaging research (e.g. Cantlon and Li 2013). The video used in the scanner was also played for each participant during their mock scanner session to minimize between-participant effects of stimulus novelty.

### MRI data acquisition

All neuroimaging data were acquired at the Alberta Children’s Hospital using a 3.0 T GE MR750w (Waukesha, WI) scanner with a 32-channel head coil. Structural images were acquired with a *T*_1_-weighted 3D BRAVO sequence with the following parameters: TR = 6.764 ms, TE = 2.908 ms, FA = 10°, voxel size 0.8 × 0.8 × 0.8 mm, and matrix size of 300 × 300. Functional images were acquired with a *T*_2_*-weighted gradient-echo EPI sequence with the following parameters: 34 axial slices, 433 volumes, TR = 2500 ms, TE = 30 ms, FA = 70°, voxel size of 3.5 × 3.5 × 3.5 mm, and matrix size of 64 × 64.

### Inattention and hyperactivity measures

To measure inattention and hyperactivity traits, parents completed the SNAP-IV, a Likert-type rating scale measuring ADHD behaviors based on DSM-IV criteria (Bussing et al. 2008). We used a shortened version of the SNAP-IV (the MTA SNAP-IV), which has 2 separate subscales that measure the participant’s level of inattention (9 items) or hyperactivity (9 items) (Bussing et al. 2008) and gives a final score for ADHD-hyperactive/impulsive (SNAP-H), ADHD-inattentive (SNAP-I), and ADHD-combined (SNAP-C). Scores can range from 0 to 3. A higher SNAP score indicates a higher level of ADHD-related behaviors. A parent-report SNAP score of greater than 1.2 is associated with an increased probability of concern, and scores above 1.8 are associated with a higher probability of ADHD diagnosis (Bussing et al. 2008). This study used the SNAP-I and SNAP-H scores.

### fMRI preprocessing

fMRI data were preprocessed with an in-house, customized Nipype pipeline (Gorgolewski et al. 2011). Steps in the anatomical image preprocessing include bias correction via ANTs (Tustison et al. 2014) *n4BiasFieldCorrection*, removal of skull and nonbrain tissue via ANTs *antsBrainExtraction*, normalization to the NIHPD 4.5- to 8.5-year-old asymmetrical atlas in MNI space (Fonov et al. 2009, 2011) using ANTs *antsRegistration*, tissue segmentation using ANTs *Atropos*, and AFNI (Cox 1996)’s *3dmask_tool* to erode the tissue segmentations.

The functional image preprocessing pipeline generally followed recommendations in Ciric et al. (2018) and Graff et al. (2022). Head motion parameters were first estimated with FSL *MCFLIRT* (Jenkinson et al. 2002) (following recommendations from Power et al. (2017) to estimate head motion parameters for regression prior to slice time correction). Functional data then underwent slice time correction with FSL’s *slicetimer*, and rigid body alignment was performed with FSL’s *MCFLIRT* (Jenkinson et al. 2002). Next, nonbrain tissue and skull stripping were performed with FSL’s *BET*. We generated a study-specific EPI template in MNI 2-mm voxel space, following recommendations by Huang et al. (2010), and used ANTs *antsRegistration* (Tustison et al. 2014) to warp the EPI image to this template. Tissue segmentations from the *T*_1_ image were warped to this functional image using FSL’s *FLIRT* (Jenkinson et al. 2002; Greve and Fischl 2009). Linear and quadratic trends were removed, and a high-pass filter at 0.01 Hz was applied. Nuisance regressors included 6 head motion parameters, white matter, cerebrospinal fluid, and global signal. We also included the derivatives, quadratic terms, and quadratic term derivatives for each of the nuisance regressors into the regression model. Volumes with a framewise displacement exceeding 0.2 mm based on the Jenkinson criteria (Jenkinson et al. 2002) were censored (Power et al. 2012, 2014; Rohr et al. 2019). Finally, functional data were smoothed using a 8.0-mm FWHM Gaussian kernel, as previous studies have suggested that the optimal smoothing kernel for ISC data is slightly larger than twice the size of the voxels (Pajula and Tohka 2014; Nastase et al. 2019).

### ISC analyses

Second-level group analyses were conducted using AFNI. Voxelwise, whole-brain ISCs were computed by calculating the Pearson correlation of the BOLD signal time course at corresponding voxels between each pair of subjects (Hasson et al. 2004). Pairwise *r*-maps were then entered into a linear mixed effects model with crossed random effects to address the statistical nonindependence in ISC data (Chen et al. 2016, 2017).

The pairwise inattention and hyperactivity terms included in models were calculated as the average SNAP-I and SNAP-H scores for each pair. When considering pairwise data for ISC analysis, it is possible to characterize the behavioral distance between individuals in a number of ways; typically, either by averaging the scores of a pair or calculating the absolute difference in scores between individuals in each pair. We selected the former metric as we are testing the hypothesis that idiosyncrasy of brain response increases with symptom scores (Finn et al. 2020). In other words, we expected that 2 children with low scores would show greater ISC to one another than 2 children with high scores, even if both pairs had the same absolute difference in scores between them. This hypothesis is grounded in the idea that children with high symptom scores will be more distractable and therefore less likely to be attentive to the shared stimulus, and that it is unlikely that individuals will be distracted in a systematic way. Similarly, we included the average age of each pair as a control covariate, as distractibility decreases with age in childhood (Hoyer et al. 2021). To account for head motion, we included the total number of censored volumes per pair of subjects as a nuisance covariate. Sex was controlled for by including dummy covariates in the model representing sex makeup of each pair (female–female, female–male, and male–male). All continuous variables and covariates were mean-centered.

We created 3 models in total: (i) one that included inattention, (ii) one that included hyperactivity, and (iii) one that included both inattention and hyperactivity as variables in the model to account for both simultaneously. This allowed us to calculate a contrast for comparing associations between these measures. Statistical significance was determined through cluster-based thresholding using a voxelwise threshold of *P* < 0.001 and a cluster forming threshold corresponding to *α* = 0.05 (Cox 1996). Figures visualizing surface projections of results were created using BrainNet Viewer (Xia et al. 2013).

To account for potential differences in attention to the stimulus, we calculated versions of all the models that included frontal eye field (FEF) synchrony as a control covariate. FEF signal has been used in previous studies as an approximate for visual attention and gaze behavior (Redcay et al. 2010; Moraczewski et al. 2018). We averaged the ISC values in all voxels within a bilateral FEF region of interest defined from the MIST parcellation (Urchs et al. 2019) for each pair and included this value as a covariate in the models.

## Results

### Sample characteristics

**Fig. 1:**
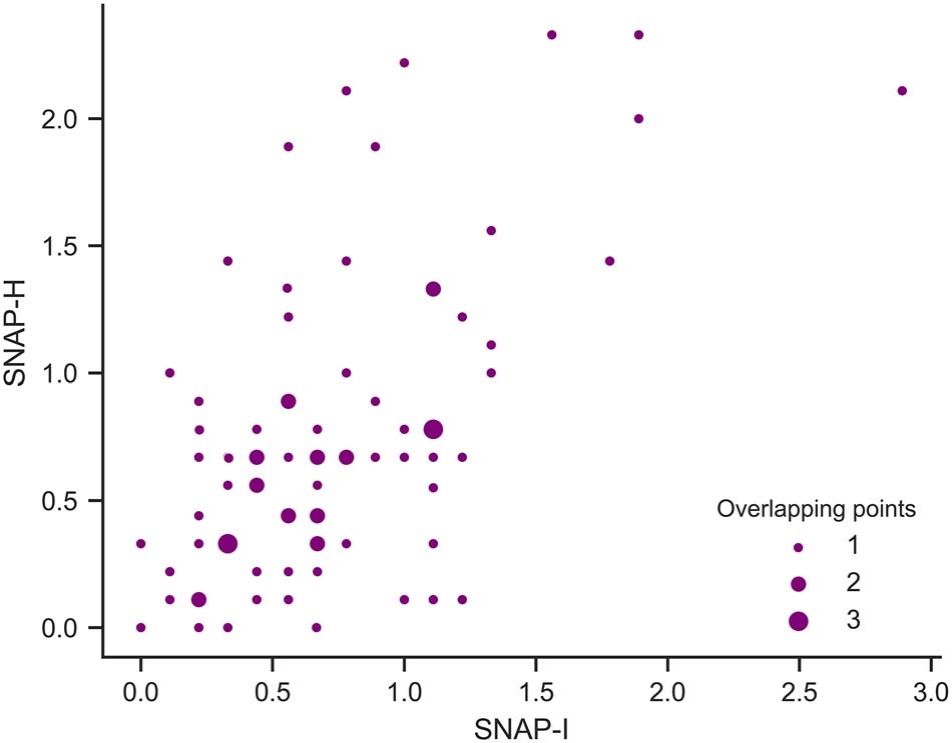
Scatterplot of SNAP-I and SNAP-H scores. The size of points on the graph denotes the number of individuals located at the coordinates to capture overlapping data points. SNAP-I and SNAP-H are correlated at Spearman’s ρ = 0.612, *P* < 0.001.

Demographic data for the final sample can be found in Table 1 and SNAP scores in Fig. 1.

### ISCs across the whole sample

**Fig. 2:**
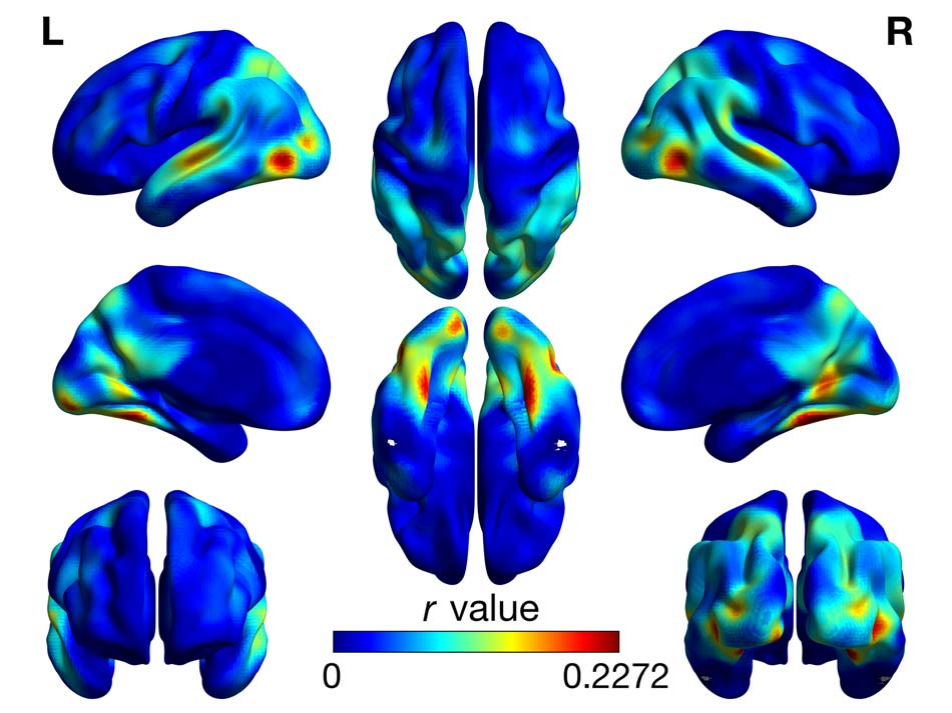
Average groupwise ISC for the whole sample. Figure shows the unthresholded Pearson’s *r* values for the whole-sample, average ISC.

Significant ISC is seen in the entire sample across almost all of the brain. Due to high statistical power from the large number of pairs included in the analysis, nearly all gray matter voxels survive cluster thresholding. Figure 2 shows the unthresholded map of group average ISC (Pearson’s *r*) of the whole sample.

### Inattention model results

**Fig. 3:**
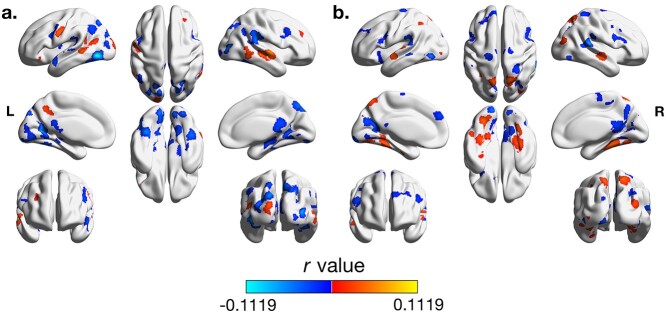
Associations between ISC and average pairwise inattentive and hyperactive trait scores. a) The associations between inattention and ISC. b) The associations between hyperactivity and ISC. Color gradient indicates the β values, in units of Pearson’s *r.* Cool colors denote negative associations (where ISC decreases as average trait score per pair increases) and warm colors denote positive associations (where ISC increases as average trait score per pair increases). For both inattention and hyperactivity, associations were seen in distributed areas of the occipital, temporal, parietal, and frontal lobes. Images are thresholded at a voxelwise threshold of *P* < 0.001 and a cluster-forming threshold of *α* = 0.05.

Higher ISC was associated with lower pairwise inattention scores in a number of areas in the occipital, temporal, and frontal cortices, including bilateral lateral occipital cortex, occipital pole, fusiform cortex, lingual gyri, supramarginal gyri, angular gyri, precuneus, precentral gyrus, parietal operculum cortex, and superior cerebellum; the right middle frontal gyrus and posterior cingulate; and the left temporal cortex, including the superior temporal gyrus and the medial temporal cortex (Fig. 3a; detailed cluster information can be found in Supplementary Table S2). Most associations were found in areas of the brain where the average group synchrony was relatively high (i.e. greater than *r* = 0.05; see Fig. 2).

Higher ISC was associated with greater pairwise inattention scores in the bilateral middle temporal gyri; the anterior right superior temporal gyrus and frontal pole; and the left occipital pole, lateral occipital cortex, supramarginal gyrus, temporal pole, orbital frontal cortex, and precentral gyrus. Models that controlled for FEF synchrony show similar results (Supplementary Fig. S1).

For all models, main findings remain generally unchanged after inclusion of the FEF covariate. Findings from these models are reported in Supplementary Figs. S1–S3.

### Hyperactivity model results

Greater ISC was associated with decreased pairwise hyperactivity scores in bilateral visual occipital cortex, precentral gyri, middle frontal gyri, middle and superior temporal gyri, and superior cerebellum; right precuneus, posterior cingulate, intracalcarine cortex, postcentral gyrus, and superior parietal lobule; and left occipital pole, inferior and superior frontal gyri, supramarginal gyrus, frontal orbital cortex, and parietal operculum cortex (Fig. 3b; detailed cluster information can be found in Supplementary Table S3).

Greater ISC was associated with elevated pairwise hyperactivity scores in bilateral visual occipital cortex, superior temporal gyrus, fusiform gyri, lingual gyri, and superior cerebellum; right middle temporal gyrus; and left inferior temporal gyrus. Models controlling for FEF synchrony showed similar results to the original model (Supplementary Fig. S2).

### Differences between inattention and hyperactivity

**Fig. 4:**
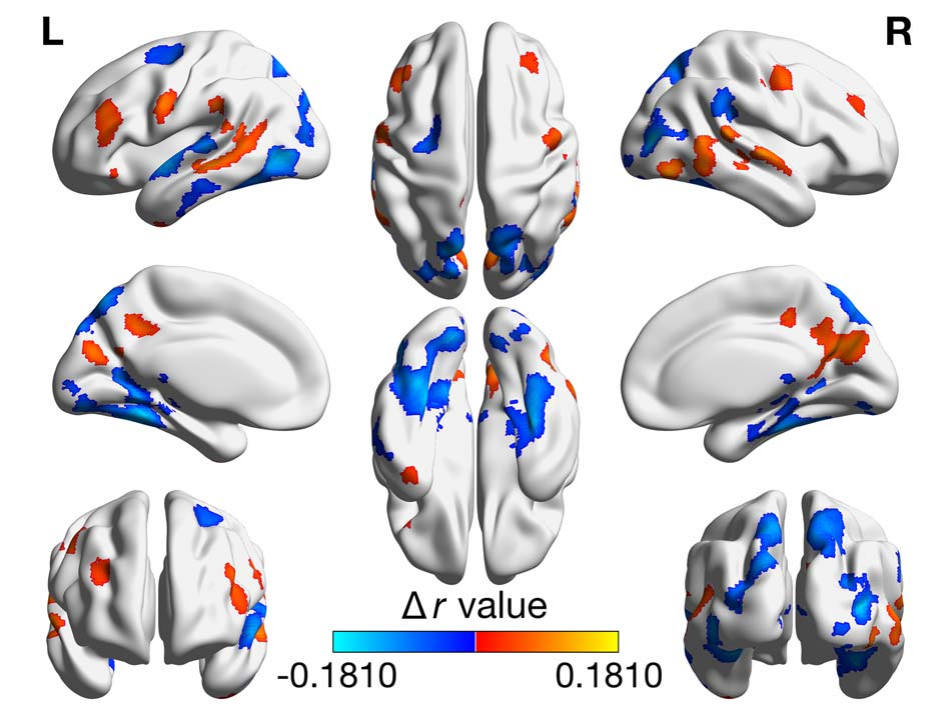
Contrast between inattention and hyperactivity. Figure shows the difference in β values, in units of Pearson’s *r*, corresponding to the inattention–hyperactivity contrast. Negative (cool) clusters indicate that pairwise inattention scores had a more negative/less positive association than pairwise hyperactivity scores, and positive (warm) clusters indicate that pairwise hyperactivity scores had a more negative/less positive association than pairwise inattention scores. Results are thresholded at a voxelwise threshold of *P* < 0.001 and a cluster-forming threshold of *α* = 0.05.

To determine whether there were dissociable relationships between ISC and inattention relative to hyperactivity, we created a third model that included both pairwise average inattention scores and pairwise average hyperactivity scores as variables. The contrast was calculated as inattention–hyperactivity, so that negative clusters are where the β for inattention scores was more negative than the β for hyperactivity scores, and vice versa for positive clusters (Fig. 4; detailed cluster information can be found in Supplementary Table S4). Pairwise inattention score had a more negative/less positive association with ISC in bilateral fusiform cortex and lateral occipital cortex; left inferior and superior gyri and supplementary motor area; and right temporoparietal junction. Hyperactivity score had a more negative/less positive association with ISC in bilateral precuneus, precentral gyrus, middle temporal gyrus, and middle frontal gyrus; right superior gyrus; and left inferior frontal gyrus.

## Discussion

This study investigated whether normative variation in inattentive and hyperactive traits in young children relates to interindividual brain synchrony during video viewing. We found that both average pairwise inattention and hyperactivity scores, as measured by the SNAP-IV, show associations with interindividual synchrony in areas engaged by the video viewing task. For inattention, negative associations—where ISC was lower in pairs with higher average trait scores—were most notably found in both dorsal and ventral visual streams, temporal auditory processing areas, bilateral temporoparietal regions, and precuneus. Hyperactivity scores had a negative association with ISC in several areas including bilateral parietal association cortex, prefrontal cortex, and precuneus. Contrary to our hypothesis of primarily negative associations between ISC and trait scores, we found regions with significant positive associations for both inattention and hyperactivity. Our findings suggest that children’s neural processing of complex audiovisual stimuli is associated with inattentive and hyperactive traits and highlight the importance of considering these traits separately and dimensionally in developmental neuroimaging research.

### Relationships between inattention/hyperactivity and the brain in typically developing populations

Population-based studies have shown that across the spectrum of ADHD traits, subclinical symptoms of ADHD in childhood are linked to poorer academic performance at age 12 (Salla et al. 2016) and 16 (Sayal et al. 2015), as well as increased grade retention and failure to graduate (Galéra et al. 2009; Bussing et al. 2010). Higher inattention scores at 6 years old were also associated with a lower annual income 3 decades later (Vergunst et al. 2019). Despite the clear implication that subthreshold inattention and hyperactivity traits might affect future outcomes, the literature characterizing the dimensional relationships between trait inattention/hyperactivity and brain measures in undiagnosed individuals (especially in early childhood) has been limited. However, there has been some evidence from both population-based studies, as well as those specifically focusing on control samples with no reported diagnoses, that inattentive and hyperactive symptomology are related to brain structure and function in nonclinical cohorts. Measures such as cortical thinning (Shaw et al. 2011; Ducharme et al. 2012; Mous et al. 2014), putamen (Mous et al. 2015) and prefrontal gray matter volume (Albaugh et al. 2017, 2019), and functional connectivity (Hilger and Fiebach 2019; Rohr et al. 2019) have shown associations with inattention and/or hyperactivity in both children and adults in the general population and nonclinical samples. While none of these studies focused specifically on ISC, they are consistent with our finding of a relationship between the brain and normative attentive traits. We have expanded upon this previous work and shown that ISC also has an association with inattention and hyperactivity in the nonclinical population.

### Separability of the inattention and hyperactivity trait dimensions in typically developing populations

In the clinical context, it has been suggested that ADHD is made up of a general underlying ADHD factor and 2 separable dimensions of inattention and hyperactivity/impulsivity (Toplak et al. 2009; Smith et al. 2013); however, it is unclear whether the separation of the inattention and hyperactivity dimensions extends into the normative spectrum of traits. Studies suggest that in the general population, inattention and hyperactivity have differential associations with cognitive measures (Kuntsi et al. 2014), symptom trajectories (Larsson et al. 2006, 2011), and educational attainment (Pingault et al. 2011). In terms of brain measures, inattention and hyperactivity have shown associations with gray matter volume in different areas of the brain in a population-based study (Albaugh et al. 2017), and Salmi et al. (2020) found different dimensional associations between ISC and inattention and hyperactivity in their adult control group. In our study, we also found different regional associations between ISC and inattention and hyperactivity in functionally diverse areas. Pairwise inattention scores had more negative/less positive relationships with ISC in areas related to sensory processing, motor planning (Abe and Hanakawa 2009), and attention/social cognition (Krall et al. 2015; Martin et al. 2019; Wilterson et al. 2021), relative to hyperactivity. This contrasts with the areas where we saw more negative/less positive associations between ISC and hyperactivity, which were found mostly in areas of language processing (Sliwinska et al. 2012; Liégeois et al. 2014; Yen et al. 2019), executive function (Friedman and Robbins 2022), and default mode (Utevsky et al. 2014; Li et al. 2019). This suggests that inattention and hyperactivity may preferentially affect different cognitive and processing systems, adding support that they are separable concepts. We note that because our sample did not include any children with an ADHD diagnosis, our findings may not generalize to children with clinically diagnosed ADHD.

### Relationships between inattention/hyperactivity traits and interindividual synchrony

Previous work looking at ADHD and ISC in adults may also offer insight into how inattention and impulsivity relate to interindividual BOLD synchrony in the nonclinical population. In their study, Salmi et al. (2020) found that in their control group, increasing pairwise similarity in impulsivity scores was associated with greater ISC in the dorsomedial prefrontal cortex, while greater similarity in inattention scores was associated with higher ISC in a very small cluster in the precuneus. Our results—that there were widespread patterns of association between synchrony and both inattention and hyperactivity in young children without an ADHD diagnosis—reinforce the argument that the heterogeneity in inattention and hyperactivity in what researchers typically refer to as control groups in case–control studies could be reflective of a linked heterogeneity among brain structure, function, and behavioral traits. This has clear implications for conducting categorical studies of ADHD and its neural correlates.

Contrary to our hypotheses, we found several areas in both the inattention and hyperactivity analyses where pairs with higher average symptoms levels are more synchronized to one another than pairs with low average scores. For inattention scores, this included temporal auditory processing areas, early visual occipital cortex, default mode network (precuneus), and left temporoparietal junction and precentral gyrus. Pairwise hyperactivity scores showed a positive association with ISC in downstream ventral visual areas, parietal association cortex, and bilateral temporal cortex. While these results were unexpected, we speculate that certain properties of the stimulus may be more salient to specific children depending on their attentive trait scores and capture their attention in a “bottom-up” way, resulting in a more stimulus-driven, synchronized response between individuals who score high on a trait. However, this interpretation will need to be confirmed in further research ideally integrating measures of attention such as eye-tracking.

### Implications for screen media and education

Our study has implications for the use of AV media in educational settings. Recent work has suggested that interindividual synchrony (as measured with electroencephalography) can be used as a marker of “neural engagement” with an educational video stimulus, as an individual’s synchrony to the rest of the group was associated with better performance on a test of the video contents. Supporting the idea that engagement is reflected in interindividual synchrony, Song et al. (2021) found that ISC in the default mode network was higher during scenes in narrative movies that were deemed more “engaging” through participant self-report. Our findings, that both inattention and hyperactivity demonstrated associations with ISC, suggest that children’s engagement with educational video stimuli may vary with their inattentive and hyperactive traits. However, as we found both negative and positive associations with ISC for both traits, we cannot yet definitively characterize the nature of these relationships—for example, both positive and negative associations with inattention scores were found in the precuneus, an area often regarded as a core node of the DMN. Due to these mixed results, it is hard to surmise whether screen media, such as the television clips used in this study, are more or less engaging for children depending on their attentive traits. Further research should explore the relationships between inattentive and hyperactive traits, interindividual synchrony, and engagement with educational videos.

### Limitations

This study had several strengths, including an early childhood age range, a dimensional perspective on inattentive and hyperactive traits, and a relatively long scan time (~18 min). The study also had several limitations to note. We found that children excluded for head motion had higher trait levels of inattention and hyperactivity than the children included in our sample at the trend level, potentially limiting generalizability. Though we controlled for sex in our models, our sample had more females than males, which is not necessarily representative of the general population, where sex has a significant effect on type, prevalence, and severity of ADHD symptoms (Smidts and Oosterlaan 2007; Arnett et al. 2015). Finally, we were not able to collect accurate eye-tracking data, so we were unable to directly assess the impact of important confounds such as gaze behavior and visual attention to the stimulus. To partially mitigate this, we calculated models that used FEF synchrony as a covariate to approximate difference in visual attention and gaze behavior between individuals, as has been done in previous work (Redcay et al. 2010; Moraczewski et al. 2018). While the general findings remain unchanged after this control analysis, we cannot definitively conclude that patterns of visual attention to the stimulus were not a driving factor in the associations we found between inattention/hyperactivity and ISC. Further work needs to be done that investigates the extent to which visual attention and gaze behavior contribute to ISC.

## Conclusions

This study found that inattentive and hyperactive traits are differentially associated with interindividual BOLD signal synchrony during the neural processing of videos by young children. An important future direction will be to ascertain whether these differences are linked with differences in understanding and retention, which could have implications for early childhood education. This work adds to a growing body of literature suggesting meaningful trait-linked variation in brain function in samples with no reported diagnoses.

## Supplementary Material

TanseySupplement_FinalProof_Feb24-2022_tgac011
