# Supplementary material for: Inattentive and hyperactive traits differentially associate with interindividual functional synchrony during video viewing in young children without ADHD
Source: Cereb Cortex Commun. 2022 Feb 28;3(1):tgac011. doi: 10.1093/texcom/tgac011 (PMC8919299; doi:10.1093/texcom/tgac011)
Supplement: TanseySupplement_FinalProof_Feb24-2022_tgac011 [file tanseysupplement_finalproof_feb24-2022_tgac011.zip › TanseySupplement_FinalProof_Feb24-2022_tgac011.docx]

**SUPPLEMENTARY MATERIAL**

*Supplementary Tables*

**Supplementary Table S1. Included vs. excluded participant demographics.** Comparison of demographics between children included and excluded from analysis (included *n* = 81; excluded *n* = 54). Children were excluded if they had less than 16 minutes (384 volumes) of non-motion corrupted data. A motion-corrupted volume was defined as a volume with a framewise displacement greater than 0.2 mm (Jenkinson et al. 2002).

|  | Age (years) | Censored volumes | Average relative FD (mm) | FSIQ | SNAP-I | SNAP-H | Sex |
| --- | --- | --- | --- | --- | --- | --- | --- |
| Included (SD) | 5.88 (0.94) | 21.32 (16.69) | 0.076 (0.030) | 111.1 (12.77) | 0.73 (0.49) | 0.76 (0.59) | F = 51  M = 30 |
| Excluded (SD) | 5.58 (0.91) | 123.28 (68.60) | 0.337 (0.357) | 106.87 (12.77) | 0.85 (0.49) | 0.95 (0.63) | F = 28  M = 26 |
| Test-statistic | *t*_53_ = 1.861 | *t*_53_ = -12.840 | *t*_53_ = -6.559 | *t*_53_ = 1.859 | *t*_53_ = -1.451 | *t*_53_ = -1.761 | *χ*^2^_1_ = 1.648 |
| *p*-value | 0.065 | < 0.001 | < 0.001 | 0.065 | 0.149 | 0.081 | 0.199 |

**Supplementary table S2. Inattention cluster details.** MNI coordinates of cluster peaks and subpeaks are reported in right-anterior-inferior (RAI) orientation.

| **Cluster size** | **Peak β** | ***t*-stat** | **x** | **y** | **z** | **Brain Regions** |
| --- | --- | --- | --- | --- | --- | --- |
| 13284 | -0.112 | -12.990 | 44 | 70 | -6 | Left lateral occipital cortex |
|  | -0.101 | -12.862 | -14 | 80 | -24 | Right Crus I |
|  | -0.076 | -12.079 | 22 | 62 | 8 | Left intracalcarine cortex |
|  | -0.084 | -11.353 | 14 | 86 | -28 | Left Crus I |
|  | -0.078 | -11.230 | -36 | 82 | 6 | Right lateral occipital cortex |
|  | -0.093 | -11.072 | -28 | 62 | -26 | Right VI |
|  | -0.068 | -9.899 | -22 | 54 | 10 | Right precuneus |
|  | -0.062 | -9.600 | 16 | 88 | 32 | Left lateral occipital cortex |
|  | -0.057 | -7.988 | 36 | 56 | -18 | Left temporal occipital fusiform cortex |
|  | -0.045 | -7.383 | 10 | 76 | -10 | Left lingual gyrus |
|  | -0.048 | -7.280 | -26 | 88 | -18 | Right occipital fusiform cortex |
|  | -0.050 | -6.387 | -24 | 94 | -2 | Right occipital pole |
|  | -0.022 | -4.326 | 32 | 94 | 22 | Left occipital pole |
|  | -0.021 | -3.830 | -12 | 72 | 0 | Right lingual gyrus |
| 1227 | -0.065 | -9.054 | -56 | 34 | 24 | Right parietal operculum cortex |
|  | -0.034 | -5.604 | -64 | 46 | 30 | Right angular gyrus |
| 674 | 0.067 | 8.405 | -58 | 12 | -2 | Right superior temporal gyrus |
| 587 | -0.041 | -8.535 | 58 | 64 | 34 | Left lateral occipital cortex |
| 586 | 0.043 | 6.471 | 24 | 56 | 40 | Left superior parietal lobule |
|  | 0.035 | 6.091 | 10 | 54 | 42 | Left precuneus |
|  | 0.031 | 4.320 | 48 | 48 | 16 | Left supramarginal gyrus |
| 563 | -0.108 | -16.874 | 68 | 26 | 2 | Left superior temporal gyrus |
|  | -0.036 | -6.978 | 68 | 44 | -2 | Left middle temporal gyrus (temporooccipital) |
|  | -0.037 | -5.868 | 54 | 30 | 20 | Left parietal operculum cortex |
|  | -0.031 | -5.041 | 50 | 32 | 38 | Left supramarginal gyrus |
|  | -0.031 | -4.980 | 40 | 36 | 12 | Left planum temporale |
| 495 | -0.034 | -6.029 | -48 | -12 | 38 | Right middle frontal gyrus |
|  | -0.025 | -4.217 | -44 | -8 | 24 | Right inferior frontal gyrus |
| 443 | 0.053 | 6.854 | 58 | 44 | 2 | Left middle temporal gyrus (temporooccipital) |
|  | 0.033 | 5.374 | 44 | 60 | 18 | Left angular gyrus |
| 417 | -0.042 | -6.994 | 46 | -4 | 22 | Left precentral gyrus |
| 376 | 0.040 | 6.130 | 16 | 92 | 16 | Left occipital pole |
| 341 | 0.043 | 6.479 | -58 | 50 | -6 | Right middle temporal gyrus (temporooccipital) |
| 321 | 0.030 | 5.650 | 38 | -22 | -32 | Left temporal pole |
|  | 0.029 | 5.139 | 48 | -22 | -6 | Left frontal orbital cortex |
| 294 | 0.032 | 5.666 | -32 | -44 | 34 | Right frontal pole |
| 256 | -0.036 | -5.337 | 52 | -2 | -10 | Left superior temporal gyrus (anterior) |
|  | -0.035 | -5.447 | 52 | -2 | -8 | Left planum polare |
| 255 | 0.042 | 6.737 | 54 | 4 | 34 | Left precentral gyrus |

**Supplementary Table S3. Hyperactivity cluster table.** MNI coordinates of cluster peaks and subpeaks are reported in right-anterior-inferior (RAI) orientation.

| **Cluster size** | **Peak β** | ***t*-stat** | **x** | **y** | **z** | **Brain Regions** |
| --- | --- | --- | --- | --- | --- | --- |
| 3812 | -0.051 | -8.434 | -24 | 64 | 2 | Right intracalcarine cortex |
|  | -0.049 | -8.228 | -20 | 54 | 14 | Right precuneus |
|  | -0.034 | -6.507 | 16 | 94 | 32 | Left occipital pole |
|  | -0.035 | -6.502 | -8 | 46 | -10 | Right I-IV |
|  | -0.034 | -5.855 | 8 | 76 | 22 | Left cuneus |
|  | -0.024 | -4.560 | -10 | 44 | 2 | Right posterior cingulate gyrus |
|  | -0.023 | -4.234 | 16 | 64 | -6 | Left lingual gyrus |
| 1630 | -0.072 | -11.226 | -14 | 88 | -22 | Right Crus I |
|  | -0.051 | -8.606 | -52 | 76 | -10 | Right lateral occipital cortex |
| 1576 | 0.047 | 6.535 | 26 | 46 | -14 | Left temporal occipital fusiform cortex |
|  | 0.045 | 7.016 | 30 | 42 | -16 | Left temporal fusiform cortex |
|  | 0.030 | 6.196 | 18 | 48 | -6 | Left lingual gyrus |
|  | 0.036 | 5.484 | 22 | 44 | -24 | Left V |
|  | 0.028 | 4.881 | 48 | 52 | -14 | Left inferior temporal gyrus |
|  | 0.022 | 3.840 | 38 | 88 | -2 | Left lateral occipital cortex |
| 900 | 0.050 | 7.094 | -28 | 40 | -16 | Right temporal occipital fusiform cortex |
|  | 0.036 | 7.772 | -22 | 46 | -2 | Right lingual gyrus |
|  | 0.038 | 5.882 | -20 | 50 | -24 | Right V |
| 801 | -0.036 | -6.504 | -48 | -8 | 26 | Right precentral gyrus |
|  | -0.030 | -5.729 | -54 | -10 | 40 | Right middle frontal gyrus |
| 775 | -0.056 | -7.680 | 12 | 94 | -12 | Left occipital pole |
|  | -0.042 | -6.609 | 28 | 80 | -28 | Left Crus I |
| 749 | -0.064 | -10.783 | 68 | 26 | 2 | Left superior temporal gyrus |
|  | -0.041 | -7.160 | 56 | 30 | 22 | Left parietal operculum cortex |
|  | -0.042 | -6.011 | 58 | 30 | -4 | Left middle temporal gyrus |
|  | -0.028 | -4.967 | 48 | 36 | 38 | Left supramarginal gyrus |
| 713 | -0.070 | -10.638 | -60 | 34 | 16 | Right planum temporal |
|  | -0.030 | -5.100 | -44 | 46 | 8 | Right middle temporal gyrus |
| 702 | 0.046 | 7.328 | 12 | 68 | 56 | Left lateral occipital cortex |
| 521 | -0.029 | -5.493 | -32 | 46 | 66 | Right superior parietal lobule |
|  | -0.016 | -3.966 | -28 | 36 | 54 | Right postcentral gyrus |
| 519 | -0.029 | -6.227 | 8 | -52 | 32 | Left superior frontal gyrus |
|  | -0.021 | -4.496 | 32 | -36 | 40 | Left middle frontal gyrus |
|  | -0.019 | -4.443 | 24 | -40 | 38 | Left frontal pole |
| 493 | 0.053 | 8.614 | -16 | 64 | 60 | Right lateral occipital cortex |
| 450 | -0.026 | -4.952 | 48 | -6 | 26 | Left inferior frontal gyrus |
|  | -0.015 | -3.771 | 62 | -8 | 24 | Left precentral gyrus |
| 354 | -0.034 | -6.144 | 20 | 22 | 66 | Left precentral gyrus |
| 315 | 0.047 | 7.040 | -56 | 14 | -8 | Right middle temporal gyrus |
|  | 0.025 | 5.970 | -48 | 6 | -18 | Right superior temporal gyrus |
| 307 | 0.042 | 6.598 | -36 | 74 | 20 | Right lateral occipital cortex |
| 287 | -0.023 | -5.689 | 28 | -16 | -14 | Left frontal orbital cortex |
| 283 | 0.048 | 6.628 | 54 | 6 | -8 | Left superior temporal gyrus |
|  | 0.037 | 5.716 | 56 | 22 | 8 | Left planum temporale |
| 238 | -0.029 | -4.424 | -10 | 14 | 68 | Right precentral gyrus |
| 237 | -0.054 | -6.757 | 42 | 70 | -6 | Left lateral occipital cortex |
| 229 | -0.032 | -5.292 | -28 | 70 | 38 | Right lateral occipital cortex |

**Supplementary Table S4. Inattention—hyperactivity contrast cluster table.** MNI coordinates of cluster peaks and subpeaks are reported in right-anterior-inferior (RAI) orientation. The contrast was calculated as inattention – hyperactivity, so that negative clusters are where the *β* for inattention was more negative/less positive than the *β* for hyperactivity, and vice versa for positive clusters.

| **Cluster size** | **Peak β** | ***t*-stat** | **x** | **y** | **z** | **Brain Regions** |
| --- | --- | --- | --- | --- | --- | --- |
| 13197 | -0.181 | -10.271 | 10 | 82 | -20 | Left occipital fusiform cortex |
|  | -0.173 | -12.231 | 24 | 46 | -12 | Left temporal occipital fusiform cortex |
|  | -0.127 | -12.203 | 48 | 82 | -12 | Left lateral occipital cortex (inferior) |
|  | -0.149 | -11.002 | -38 | 82 | 8 | Right lateral occipital cortex (inferior) |
|  | -0.130 | -10.854 | -34 | 32 | -26 | Right temporal fusiform cortex |
|  | -0.176 | -10.649 | -16 | 78 | -20 | Right VI |
|  | -0.126 | -10.613 | 18 | 52 | -4 | Left lingual gyrus |
|  | -0.151 | -10.072 | 26 | 46 | -24 | Left VI |
|  | -0.124 | -9.261 | 16 | 72 | 50 | Left lateral occipital cortex (superior) |
|  | -0.121 | -9.048 | -16 | 66 | 54 | Right lateral occipital cortex (superior) |
|  | -0.062 | -6.261 | 18 | 32 | -10 | Left parahippocampal gyrus |
|  | -0.047 | -4.780 | -52 | 46 | -28 | Right inferior temporal gyrus (temporooccipital) |
| 2354 | 0.098 | 7.958 | -10 | 66 | 24 | Right precuneus |
|  | 0.092 | 7.400 | -14 | 74 | 26 | Right cuneus |
|  | 0.075 | 7.162 | 10 | 50 | 38 | Left precuneus |
|  | 0.082 | 6.510 | 6 | 76 | 22 | Left cuneus |
| 1108 | -0.135 | -8.802 | 54 | 4 | -6 | Left superior temporal gyrus (anterior) |
|  | -0.092 | -6.887 | 48 | 28 | 6 | Left planum temporale |
|  | -0.042 | -4.147 | 42 | -8 | -32 | Left temporal pole |
| 816 | 0.070 | 6.173 | 44 | -34 | 26 | Left middle frontal gyrus |
| 740 | 0.109 | 6.855 | 58 | 28 | -4 | Left middle temporal gyrus (posterior) |
|  | 0.067 | 5.147 | 48 | 48 | 12 | Left supramarginal gyrus |
| 724 | 0.079 | 7.568 | 46 | -18 | -14 | Left temporal pole |
| 668 | 0.091 | 6.469 | -56 | 52 | -4 | Right middle temporal gyrus (temporooccipital) |
|  | 0.091 | 6.532 | -62 | 38 | 12 | Right supramarginal gyrus |
| 523 | -0.088 | -6.470 | -50 | 36 | 26 | Right parietal operculum cortex |
|  | -0.074 | -6.327 | -68 | 28 | 32 | Right supramarginal gyrus |
| 456 | 0.072 | 6.173 | -50 | 2 | 44 | Right precentral gyrus |
| 435 | 0.095 | 7.556 | 62 | 32 | 26 | Left supramarginal gyrus |
| 429 | 0.088 | 7.810 | 56 | 2 | 30 | Left precentral gyrus |
| 424 | -0.085 | -6.854 | 26 | 8 | 54 | Left precentral gyrus |
|  | -0.056 | -5.140 | 20 | -4 | 58 | Left superior frontal gyrus |
| 347 | 0.101 | 6.389 | -60 | 16 | 0 | Right superior temporal gyrus (posterior) |
|  | 0.084 | 7.934 | -64 | 0 | -14 | Right middle temporal gyrus (anterior) |
| 329 | 0.063 | 5.890 | -32 | -44 | 32 | Right frontal pole |
| 316 | 0.131 | 10.501 | -52 | 74 | -14 | Right lateral occipital cortex (inferior) |
| 315 | 0.112 | 6.799 | -30 | 76 | -32 | Right Crus I |
| 251 | 0.099 | 6.831 | 32 | 74 | -32 | Left Crus I |
| 243 | -0.055 | -4.931 | 48 | 66 | 42 | Left lateral occipital cortex (superior) |
| 233 | 0.090 | 7.225 | -42 | 44 | 42 | Right supramarginal gyrus |
| 226 | 0.085 | 6.388 | -34 | 74 | 40 | Right lateral occipital cortex (superior) |

**Supplementary Figure S1.** **Associations between ISC and inattention, model with FEF synchrony included as a control.** Colour gradient indicates the *β* values, in units of Pearson’s *r*. Cool colors denote negative associations (where ISC decreases as average trait score per pair increases) and warm colors denote positive associations (where ISC increases as average trait score per pair increases). Images are thresholded at a voxelwise threshold of *p* < 0.001 and a cluster-forming threshold of *α* = 0.05. All supplementary figures were created with BrainNet Viewer (Xia et al. 2013).

**
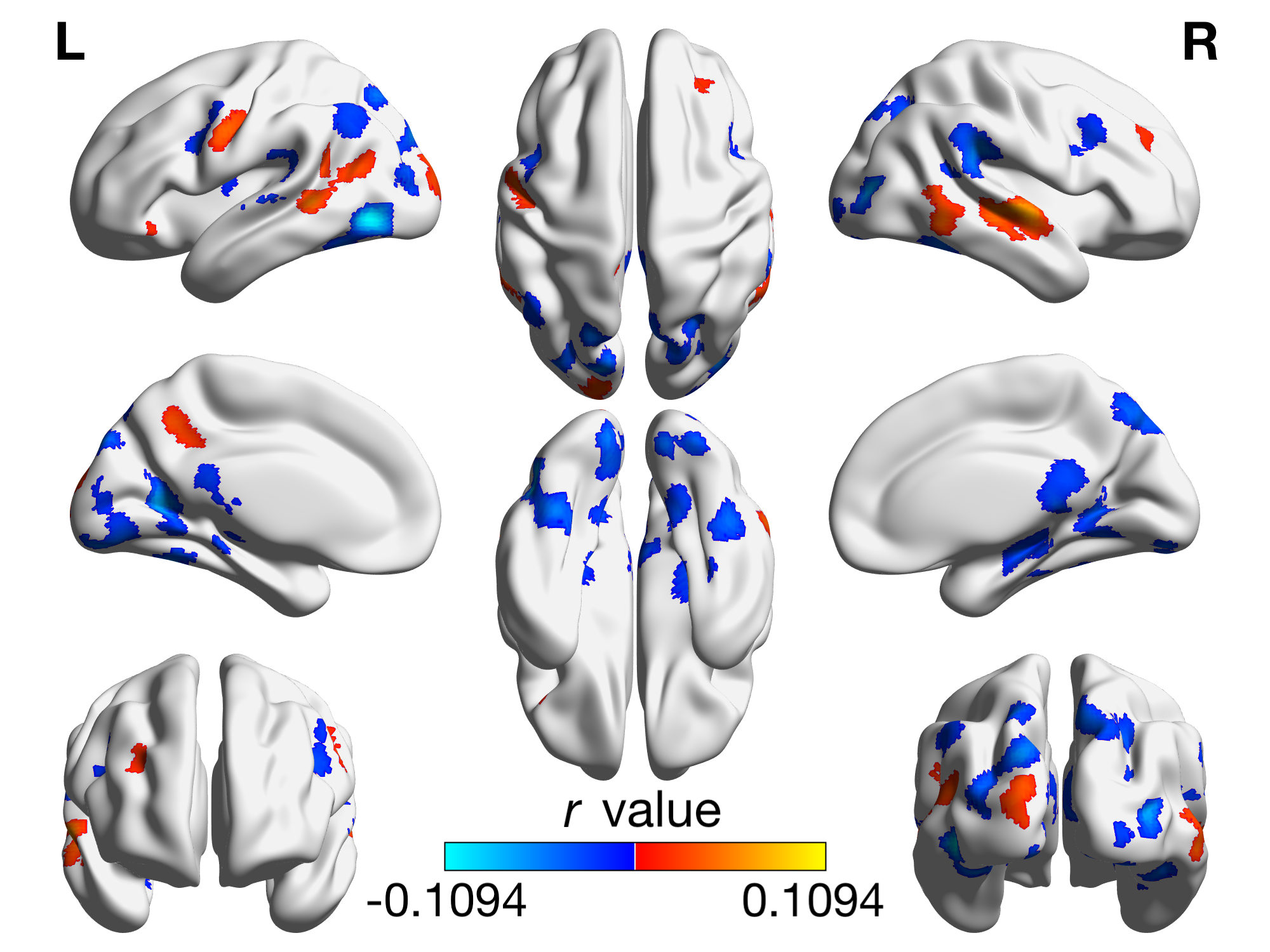
**

**Supplementary Figure S2. Associations between ISC and hyperactivity, model with FEF synchrony included as a control.** Colour gradient indicates the *β* values, in units of Pearson’s *r* Cool colors denote negative associations (where ISC decreases as average trait score per pair increases) and warm colors denote positive associations (where ISC increases as average trait score per pair increases). Images are thresholded at a voxelwise threshold of *p* < 0.001 and a cluster-forming threshold of *α* = 0.05.


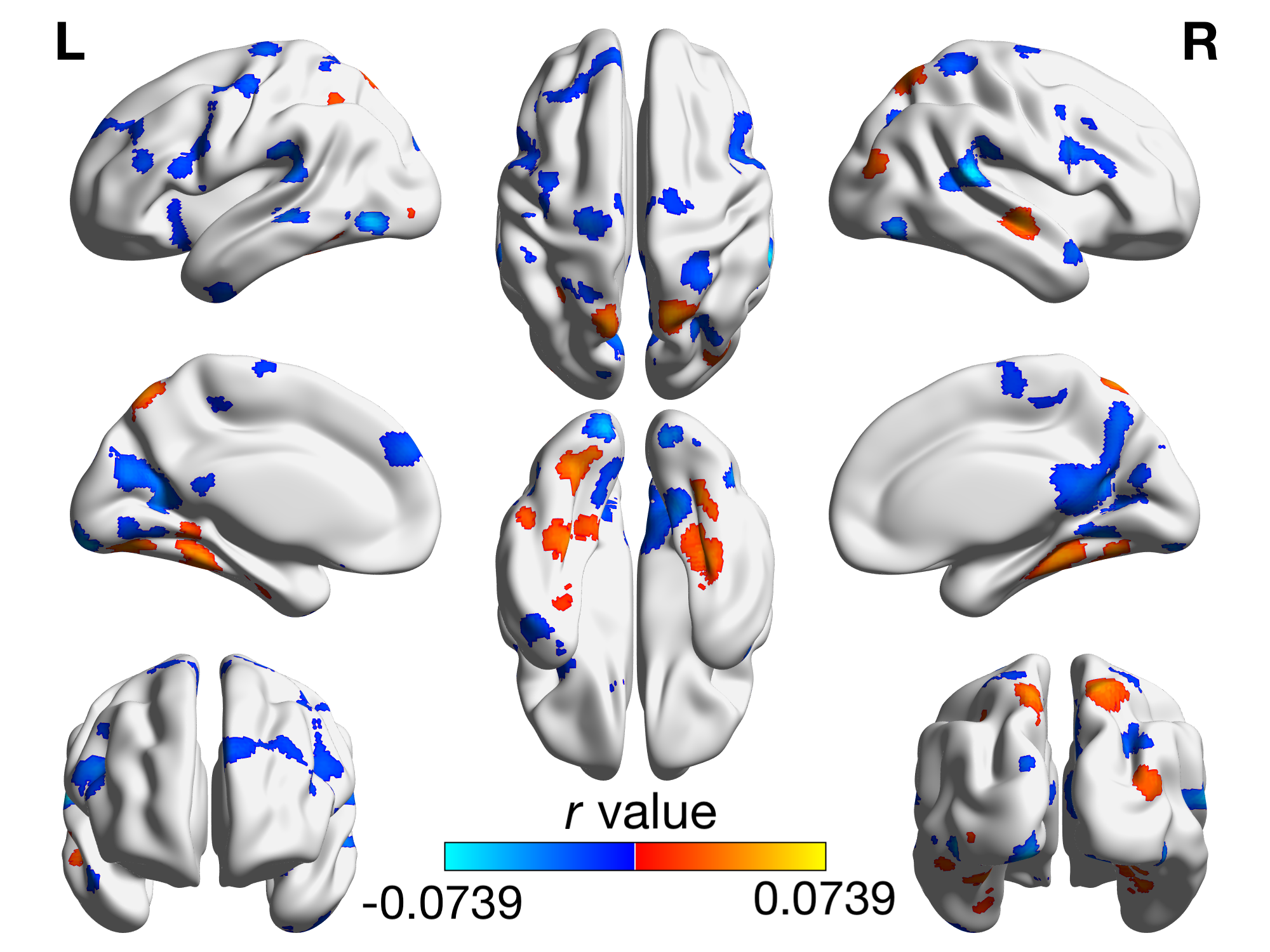


**Supplementary Figure S3. Contrast between inattention and hyperactivity covariates when included in the same LME, model with FEF synchrony included as a control.** Figure shows the difference in *β* values, in units of Pearson’s *r,* corresponding to the inattention – hyperactivity contrast. Negative (cool) clusters indicate that inattention had a more negative/less positive association than hyperactivity, and positive (warm) clusters indicate that hyperactivity had a more negative/less positive association than inattention. Results are thresholded at a voxelwise threshold of *p* < 0.001 and a cluster-forming threshold of *α* = 0.05.


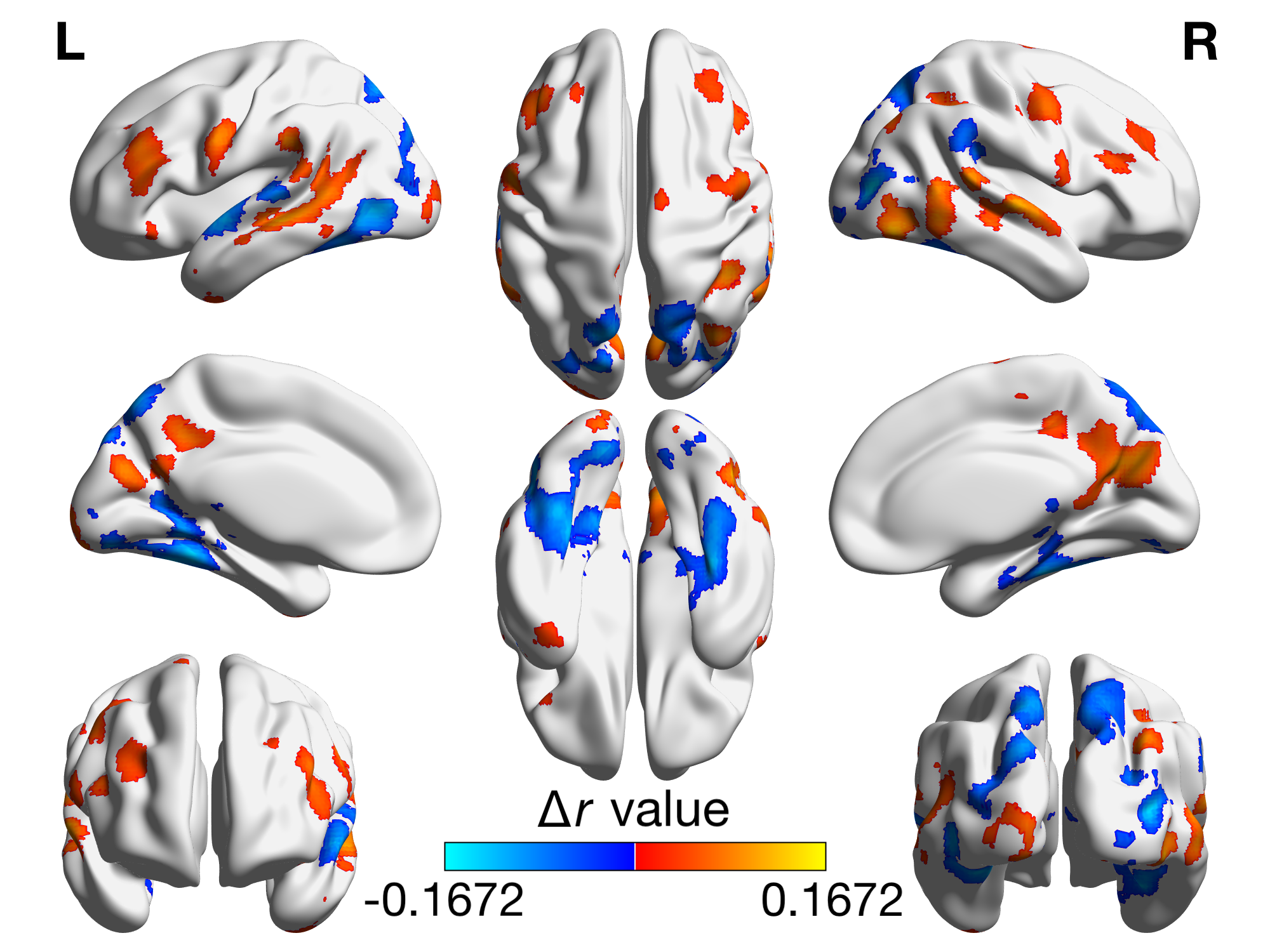


**References**

Jenkinson M, Bannister P, Brady M, Smith S. 2002. Improved optimization for the robust and accurate linear registration and motion correction of brain images. Neuroimage. 17:825–841.

Xia M, Wang J, He Y. 2013. BrainNet Viewer: A Network Visualization Tool for Human Brain Connectomics. PLoS One. 8.
